# Continued History of a Case of “Gouging”

**Published:** 1863-08

**Authors:** E. L. Holmes

**Affiliations:** Chicago, One of the Surgeons of the Chicago Charitable Eye and Ear Infirmary


### CONTINUED HISTORY OF A CASE OF "GOUGING."

By E. L. HOLMES, M. D., of Chicago,

*One of the Surgeons of the Chicago Charitable Eye and Ear Infirmary.*

In a former number of this Journal (December, 1859,) may be found the history of a case of injury of the eyes which has scarcely a parallel in the annals of Ophthalmic Science. I have lately had an opportnnity of examining the patient, and hope an account of his present condition will not be uninteresting to the reader of the *Journal.*

For the benefit of those who may have forgotten the previous history of the case, I may state that an Irishman, 37 years of age, was attacked by some one, who forced his thumbs with such violence into the patient's orbits, as to rupture both globes. For six weeks the patient remained in the southern part of this State, totally blind, and received, as he stated, no other treatment than repeated doses of Sulphate of Magnesia, internally, and wet compresses locally. At the expiration of this time he was able to distinguish light, and gradually regained indistinct vision of large objects. Five months after the assault the patient came under my care. With the right eye he could see light and the shadows of objects passed in front of the eye; with the left eye he could, with a little assistance from an attendant, conduct himself in any of the streets of Chicago. The cornea of each eye was perfectly normal in appearance. The upper and inner sixth of each iris was absent; it had apparently been torn away, as in an operation for Iridiotomy. The pupils were consequently elongated. Near the inner extremity of each pupil was a cicatrix, at the union of the sclerotied and cornea, in which were involved particles of pigment, probably from the iris. A rupture of the globes had, without doubt, occurred at this place, and through the opening had evidently been forced the lost portion of iris and crystalline lens of each eye, for in neither eye could a trace of the lens be fonnd. Trembling of the iris, usually observed after the removal of the lens, was absent in both eyes, probably on account of tension of the radiating fibres of the iris, produced by their contraction during cicatrization.

The vitreous humor of each eye was shown by the Ophthalmoscope to be so clouded with fine and black particles floating in its substance, that the vessels of the retina and the papilla of the optic nerve could be readily seen, especially in the right eye.

By the use of a simple supporting treatment the patient was so far improved in four months as to be able to walk without assistance and to read large signs across the streets, although he could not, even with the aid of double convex lenses, distinguish minute objects. The vitreous humor of both eyes had become nearly transparent, although vision of the right eye had not improved.

A few weeks since, more than three years and a half after my former report, the patient again consulted me in reference to his vision. Five months previously the perception of light in the right eye began to fail and in less than four months it was wholly extinct. The vision of the other eye has remained as before with possibly a slight improvement. The only change observable by simple inspection, is a marked trembling of each iris, which, it will be remembered, was not present three years ago.

In the right eye neither papilla nor vessels can be discovered with the Ophthalmoscope. Even the peculiar red disk almost invariably observed through the pupil by the Ophthalmoscope, cannot be seen.

By means of light concentrated by a powerful double convex lens obliquely upon the cornea, and thrown into the pupil a dark bluish mass can be seen lying behind the iris; this is undoubtedly the retina which has been detached from the choroid.

The papilla of the other eye is of an oval form and of a greenish blue color. It appears smaller than in a normal eye. The line of demarkation between the papilla and the retina is very distinct, except at its upper and outer portion, where a part of the optic nerve seems to be covered with lymph. Whether the oval form of the papilla is produced by an increase of convexity in the cornea in the direction corresponding to the long diameter of the papilla, I am unable to say.

The vessels of the retina are finer than in a normal condition of the eye, and appear more like solid threads than like vessels. The absence of the lens, which has a large magnifying power, will account for the apparent decrease in the size of the papilla and vessels. The red appearance of the retina and choroid is somewhat darker than normal, and different portions of these membranes seem of a different shade of redness.

